# Physical rehabilitation in Brazilian pediatric intensive care units: a multicenter point prevalence study

**DOI:** 10.5935/2965-2774.20230388-en

**Published:** 2023

**Authors:** Juliana Redivo, Harini Kannan, Andreia Aparecida Freitas Souza, José Colleti Junior, Sapna Ravi Kudchadkar, Acute Rehabilitation for Kids in the PICU (PARK-PICU) Investigators, Nelson Kazunobu Horigoshi, Graziela de Araújo Costa, Taísa Roberta Ramos de Castilho, Paula Peres Domingues Peron, Walter Perez Scaranto, Daniela Nasu Monteiro Medeiros, Toshio Matsumoto, Carlos Gustavo de Almeida, Felipe Rezende Caino de Oliveira, Marcelo Barciela Brandão, Fernanda Lima-Setta, Arnaldo Prata-Barbosa, Glaciele Nascimento Xavier, Livia Barbosa de Andrade, Agda Ultra de Aguiar, Marcos Paulo Galdino Coutinho, Roberta Esteves Viera de Castro, Glazia André Landy, Suzana Lopes Bonfim Balaniuc, Ricardo Silveira Yamaguchi

**Affiliations:** 1 Department of Anesthesiology and Critical Care Medicine, Charlotte R. Bloomberg Children’s Center, Johns Hopkins University School of Medicine - Baltimore, United States; 2 Department of Pediatrics, Hospital Infantil Sabará - São Paulo (SP), Brazil; 3 Department of Pediatrics, Hospital Israelita Albert Einstein - São Paulo (SP), Brazil; 4 Department of Pediatrics, Johns Hopkins University School of Medicine - Baltimore, United States; 5 Hospital Infantil Sabará, São Paulo, SP Brazil; 6 Hospital Sírio- Libanês, São Paulo, SP Brazil; 7 Hospital Anália Franco Rede D’Or São Luiz Brazil; 8 Hospital Beneficência Portuguesa, São Paulo, SP Brazil; 9 Instituto de Criança, Hospital das Clínicas, Universidade de São Paulo, São Paulo, SP Brazil; 10 Hospital Municipal Carmino Caricchio, Tatuapé, São Paulo, SP Brazil; 11 Hospital Municipal Dr. Moyses Deutch, M’Boi Mirim, São Paulo, SP Brazil; 12 Hospital Municipal Infantil Menino Jesus, São Paulo, SP Brazil; 13 Hospital Assunção Rede D’Or São Luiz, São Bernardo do Campo, SP Brazil; 14 Grupo de Apoio ao Adolescente e à Criança com Câncer, Instituto de Oncologia Pediátrica, São Paulo, SP Brazil; 15 Universidade de Campinas, Campinas, SP Brazil; 16 Instituto Fernandes Figueira, FIOCRUZ, Rio de Janeiro, RJ Brazil; 17 Hospital Copa D’Or, Rio de Janeiro, RJ, Brasil; 18 Hospital Quinta D’Or, Rio de Janeiro, RJ, Brasil; 19 Hospital Caxias D’Or, Rio de Janeiro, RJ, Brasil; 20 Hospital Rios D’Or, Rio de Janeiro, RJ, Brasil; 21 Hospital Oeste D’Or, Rio de Janeiro, RJ, Brasil; 22 Hospital Real D’Or, Rio de Janeiro, RJ, Brasil; 23 Instituto de Cardiologia do Distrito Federal, Brasília, DF, Brasil; 24 Hospital Esperança, Recife, PE, Brasil; 25 Hospital de Base do Distrito Federal, Brasília, DF Brazil; 26 Hospital Otávio de Freitas, Recife, PE, Brasil; 27 Hospital Universitário Pedro Ernesto, Universidade do Estado do Rio de Janeiro, Rio de Janeiro, RJ Brazil; 28 Instituto de Tratamento do Câncer Infantil - São Paulo, SP Brazil; 29 Hospital Universitário Maria Aparecida Pedrossian, Campo Grande, MS Brazil; 30 Hospital da Luz, São Paulo, SP Brazil

**Keywords:** Critical care, Occupational therapy, Physical therapy modalities, Rehabilitation, Intensive care units, pediatric

## Abstract

**Objective:**

To determine the prevalence and factors associated with the physical rehabilitation of critically ill children in Brazilian pediatric intensive care units.

**Methods:**

A 2-day, cross-sectional, multicenter point prevalence study comprising 27 pediatric intensive care units (out of 738) was conducted in Brazil in April and June 2019. This Brazilian study was part of a large multinational study called Prevalence of Acute Rehabilitation for Kids in the PICU (PARK-PICU). The primary outcome was the prevalence of mobility provided by physical therapy or occupational therapy. Clinical data on patient mobility, potential mobility safety events, and mobilization barriers were prospectively collected in patients admitted for ≥ 72 hours.

**Results:**

Children under the age of 3 years comprised 68% of the patient population. The prevalence of therapist-provided mobility was 74%, or 277 out of the 375 patient-days. Out-of-bed mobility was most positively associated with family presence (adjusted odds ratios 3.31;95%CI 1.70 - 6.43) and most negatively associated with arterial lines (adjusted odds ratios 0.16; 95%CI 0.05 - 0.57). Barriers to mobilization were reported on 27% of patient-days, the most common being lack of physician order (n = 18). Potential safety events occurred in 3% of all mobilization events.

**Conclusion:**

Therapist-provided mobility in Brazilian pediatric intensive care units is frequent. Family presence was high and positively associated with out-of-bed mobility. The presence of physiotherapists 24 hours a day in Brazilian pediatric intensive care units may have a substantial impact on the mobilization of critically ill children.

## INTRODUCTION

Survival rates for children who require intensive care for the treatment of life-threatening illnesses or injuries have dramatically improved worldwide. The vast majority of children survive critical illness, but there is increasing evidence of pediatric intensive care unit (ICU)-acquired complications that impact patients’ shortand long-term function.^(1-4)^ Survivors of critical illness commonly experience long-term physical, cognitive, and psychological morbidities, known as post-intensive care syndrome.^(3,5)^ Thus, there is growing clinical and research interest in physical rehabilitation interventions initiated in the pediatric ICU that may prevent these complications and optimize functional outcomes in critically ill children.^(6)^

Early rehabilitation and mobility in adult ICUs are associated with improved muscle strength and physical functioning, along with decreased mechanical ventilation duration.^(7,8)^ In 2010, the *Associação de Medicina Intensiva Brasileira* (AMIB released Guidelines for Early Mobilization in Intensive Care Unit.^(9)^ Despite this renewed focus on acute rehabilitation, there are few studies on early mobilization in the ICU in Brazil. Data from adult ICUs in Brazil show that the prevalence of patient mobilization is variable; however, few mechanically ventilated patients with an endotracheal tube are mobilized out of bed as part of routine care.^(10-12)^ Although there are more than 5,000 registered pediatric ICU beds in Brazil, there is a lack of data regarding the current state of rehabilitation practices for infants and children who are undergoing active neurocognitive and physical development.^(13)^ Previous point prevalence studies of pediatric ICU rehabilitation in the USA and Europe have shown that early rehabilitation consultation is infrequent, and endotracheal tube use is negatively associated with out-of-bed mobility.^(14,15)^ Brazilian pediatric ICU practices and staffing differ from these regions, as the physiotherapists provide both physical and respiratory therapy.

Thus, we conducted a 2-day point prevalence study in 27 pediatric ICUs across Brazil as part of a multinational study called the Prevalence of Rehabilitation for Kids in the PICU (PARK-PICU), a collaboration with the Investigators and the Pediatric Acute Lung Injury and Sepsis Investigators (PALISI) network.^(14)^ The primary objective was to determine the prevalence of physical rehabilitation and mobility for patients admitted for at least 3 days. Additionally, we evaluated perceived barriers and potential safety events for patient mobility.

## METHODS

The PARK-PICU was a cross-sectional point prevalence study conducted in different regions of the world to characterize rehabilitation practices for pediatric ICU patients. Full details of the PARK-PICU methodology are described in detail elsewhere,^(16)^ and the study was conducted in Brazil using the same exact methodology and inclusion criteria. In Brazil, 27 pediatric ICUs comprising 316 beds (out of 738 Brazilian pediatric ICUs, comprising 9,536 beds)^(13)^ participated on 2 days (April 16, 2019, and June 6, 2019). Pediatric ICUs in Brazil were eligible to participate if they cared for mechanically ventilated infants and children and were located in a distinct physical space dedicated to pediatric patients. Pediatric ICUs were recruited via email by AMIBnet, the research branch of the AMIB. Site principal investigators were instructed to complete the pediatric ICU organizational survey in collaboration with their multiprofessional team to ensure the accuracy of responses to all items. Institutional review board approval was obtained at all participating sites (CAAE 89274218.7.1001.5458).

### Electronic case report forms

The entire REDCap platform was translated to Portuguese specifically to facilitate the PARK-PICU study in Brazil. All data collection forms were adapted from those used in the PARK-PICU USA study. Bedside data collection forms (e.g., activity events) were also translated to Portuguese and are available on the study website.^(16)^

### Data analysis/statistical methods

The prevalence of therapist-provided mobility was defined as the number of patient-days in which a physical therapist (PT) or occupational therapy (OT) was involved in mobilizing a patient divided by the total number of patient-days. Activities that were classified as out-of-bed were as follows: being held by a parent or nurse, transfer from bed to chair, standing, marching or walking in the room or unit, and walking off the unit.

To analyze categorical data, the chi-squared test was utilized. Continuous data are expressed herein as the median (interquartile range - IQR) and were analyzed using the Mann‒Whitney U test. Patients who stayed in the pediatric ICU < 72 hours or who were discharged before 12 pm on the study day were excluded. Multivariable logistic regression models, adjusted with a randomized effect for ICU sites, were used to calculate adjusted odds ratios (aORs) with 95% confidence intervals (95%CIs) for therapist-provided mobility and out-of-bed mobility. Covariates were chosen based on clinical relevance and previous studies. Statistical significance was assigned to two-tailed p values of less than 0.05. Stata 16 software (StataCorp LLC, College Station, TX) was used for all statistical analyses.

## RESULTS

### Intensive care unit characteristics

Table 1 displays the pediatric ICU characteristics. Most hospitals are academic (55%), but only 44% have an early mobilization protocol. Of all participating pediatric ICUs (n = 27), 48% (n = 13) were medical-surgical-cardiac, 48% (n = 13) were medical surgical, and 4% (n = 1) were cardiac units. The median number of beds was 10 (IQR 7 - 16). A request for a therapist consultation was required in 81% of all units for therapist involvement (n = 25). At least one dedicated PT was present in 89% of all pediatric ICUs, whereas only 11% of units had a dedicated OT.

**Table 1:** Pediatric intensive care unit characteristics

Characteristics	
Academic teaching hospital	15 (55)
Freestanding children’s hospital	7 (26)
Type of pediatric ICU	
Medical-surgical cardiac	13 (48)
Medical-surgical	13 (48)
Cardiac	1 (4)
Number of beds	10 (7 - 16)
*Delirium* screening protocol	6 (22)
Early mobilization protocol	12 (44)
Dedicated physical therapist	24 (89)
Dedicated occupational therapist	3 (11)

ICU - intensive care unit. The results are expressed as n (%) or median (interquartile range).

### Patient baseline characteristics

Patient baseline characteristics are shown in table 2. Over the 2 study days, 375 patients met the inclusion criteria. Seven percent (25/375) of patients had records for both days. Most patients (68%, 256/375) were less than 3 years old, and 57% (212/375) of patients were male. The median pediatric ICU length of stay on the study day was 10 days (IQR 5 - 25). Sixty-three percent (236/375) of patients had good or mild disability in baseline function (Pediatric Cerebral Performance Category - PCPC score < 3), and 86% (321/375) were medical patients.

**Table 2:** Patient baseline characteristics by physical therapy/occupational therapy-provided mobility on the study day

Characteristics	All patient-days n = 375	PT/OT-provided mobility n = 277	No PT/OT-provided mobility n = 98	p value
Age				0.022
0 - 2	256 (68)	178 (64)	78 (80)	
3 - 6	55 (15)	48 (17)	7 (7)	
7 - 12	47 (13)	36 (13)	11 (11)	
13 - 18	17 (5)	15 (5)	2 (2)	
> 18	0	0	0	
Gender, male	212 (57)	159 (57)	53 (54)	0.569
Ethnicity				0.038
White	248 (66)	172 (62)	76 (78)	
Black	31 (8)	24 (9)	7 (7)	
Asian	1 (0)	1 (0)	0 (0)	
Brown	95 (25)	80 (29)	15 (15)	
Other	0	0	0	
BMI	16 (14 - 19)	16 (14 - 19)	16 (14 - 18)	0.0261
Pediatric Cerebral Performance Category				< 0.001
Good	171 (46)	112 (40)	59 (60)	
Mild disability	65 (17)	48 (17)	17 (17)	
Moderate disability	43 (11)	30 (11)	13 (13)	
Severe disability	78 (21)	73 (26)	5 (5)	
Coma/vegetative state	18 (5)	14 (5)	4 (4)	
Ambulatory prior to admission (data for age ≥ 3)	65 (56)	53 (55)	12 (63)	0.494
Primary reason for ICU admission				0.089
Surgical				
Neurologic	18 (5)	16 (6)	2 (2)	
Cardiac	14 (4)	11 (4)	3 (3)	
Orthopedic	2 (1)	1 (0)	1 (1)	
Pediatric surgery	13 (3)	10 (4)	3 (3)	
Other	7 (2)	6 (2)	1 (1)	
Medical				
Hematology-oncology	11 (3)	8 (3)	3 (3)	
Cardiac	16 (4)	11 (4)	5 (5)	
Infectious/inflammatory	41 (11)	34 (12)	7 (7)	
Neurologic	30 (8)	25 (9)	5 (5)	
Renal	10 (3)	8 (3)	2 (2)	
Respiratory	191 (51)	134 (48)	57 (58)	
Trauma	2 (1)	0 (0)	2 (2)	
Gastrointestinal	14 (4)	7 (3)	3 (7)	
Other^*^	6 (2)	6 (2)	0 (0)	
Admission source				0.520
Emergency room	184 (49)	134 (48)	50 (51)	
Floor/step-down unit	57 (15)	37 (13)	20 (20)	
Outside hospital	83 (22)	64 (23)	19 (19)	
OR/post-anesthesia	23 (6)	19 (7)	4 (4)	
Neonatal ICU	16 (4)	13 (5)	3 (3)	
Rehabilitation facility	2 (1)	2 (1)	0 (0)	
Other†	10 (3)	8 (3)	2 (2)	
Hospital day	12 (6 - 33)	15 (7 - 40)	8.5 (5 - 16)	0.0531
Pediatric ICU days	10 (5 - 25)	12 (6 - 33)	8 (5 - 14)	0.0637
Surgery during pediatric ICU stay, yes	109 (29)	86 (31)	23 (23)	0.150
Post-op day	12 (5 - 23)	13 (6 - 26)	8 (4 - 14)	0.0512

PT - physical therapy; OT - occupational therapy; BMI - body mass index ICU - intensive care unit.

* Includes anaphylaxis, diabetic ketoacidosis, electrolyte imbalance, exogenous intoxication, steroid treatment; † includes home care. The results are expressed as n (%) or median (interquartile range).

### Patient clinical characteristics

Mechanically ventilated patients comprised 39% of all patients (via endotracheal tube or tracheostomy). Thirty-one percent of patients had continuous sedation, 11% received a vasoactive infusion, and 55% of patients had a central venous catheter. Family was present at the bedside for 82% of the patients. Other patient clinical characteristics and support (such as lines, tubes, and extracorporeal membrane oxygenation - ECMO) are displayed in table 3.

**Table 3:** Patient clinical characteristics on the study day, by physical therapy/occupational therapy-provided mobility status

	All patient-days n = 375	PT/OT-provided mobility n = 277	No PT/OT-provided mobility n = 98	p value
Respiratory support				0.003
None	114 (30)	87 (31)	27 (28)	
Nasal cannula or face mask	54 (14)	38 (14)	16 (16)	
HFNC	23 (6)	14 (5)	9 (9)	
CPAP or BiPAP	36 (10)	26 (9)	10 (10)	
Mechanical ventilation - ETT	90 (24)	58 (21)	4 (4)	
Mechanical ventilation - Trach	58 (15)	54 (19)	32 (33)	
Mechanical ventilation characteristics				
Conventional ventilation	144 (99)	110 (100)	34 (94)	
HFOV	2 (1)	0	2 (6)	
FiO_2_	30 (25 - 40)	30 (25 - 40)	30 (30 - 40)	0.020
PEEP	7 (6 - 7)	7 (6 - 7)	6 (5 - 7)	0.030
Any continuous sedation	117 (31)	80 (29)	37 (38)	< 0.001
Opiate	75 (20)	50 (18)	25 (26)	< 0.001
Benzodiazepine	82 (22)	61 (22)	21 (21)	< 0.001
Alpha agonist	31 (8)	19 (7)	12 (12)	0.001
Barbiturate	7 (2)	7 (3)	0	0.33
Propofol	1 (0)	1 (0)	0	0.22
Ketamine	24 (6)	16 (6)	8 (8)	< 0.001
Sedation score measured	214 (14)	179 (13)	35 (18)	0.002
SBS	1 (0)	0	1 (3)	
RASS	138 (64)	133 (74)	5 (14)	
Ramsay	2 (1)	1 (1)	1 (3)	
Comfort	70 (33)	41 (23)	29 (81)	
Other (BIS)	4 (2)	4 (2)	0	
GCS	13 (8 - 15)	13 (8-15)	14.5 (9 - 15)	< 0.001
Delirium measured	75 (5)	74 (5)	1 (1)	0.972
^*^Delirium positive	10 (3)	9 (3)	1 (1)	
Family present at bedside	306 (82)	225 (81)	81 (83)	0.754
Nurse to patient ratio				< 0.001
1 to 1	47 (13)	23 (8)	24 (24)	
1 to 2	277 (74)	219 (79)	58 (59)	
1 to 3	51 (14)	35 (13)	16 (16)	
2 to 1	0	0	0	
Antipsychotics	26 (7)	18 (7)	8 (8)	0.577
Risperidone	10 (3)	7 (3)	3 (3)	0.778
Quetiapine	2 (1)	1 (0)	1 (1)	0.441
Olanzapine	0	0	0	NA
Haloperidol	5 (1)	3 (1)	2 (2)	0.477
Other	11 (3)	8 (3)	3 (3)	0.930
At least one vasoactive drug	43 (11)	33 (12)	10 (10)	0.648
Milrinone	11 (3)	7 (3)	4 (4)	0.433
Epinephrine	19 (5)	16 (6)	3 (3)	0.292
Dopamine	2 (1)	2 (1)	0	0.399
Norepinephrine	17 (5)	13 (5)	4 (4)	0.803
Vasopressin	1 (0)	1 (0)	0	0.551
Phenylephrine	0	0	0	NA
Dobutamine	9 (2)	7 (3)	2 (2)	0.787
Other (sodium nitroprusside)	1 (0)	1 (0)	0	0.551
Central line	206 (55)	143 (52)	63 (64)	0.030
Femoral	24 (6)	19 (7)	5 (5)	0.541
Neck	89 (24)	58 (21)	31 (32)	0.032
Subclavian	35 (9)	28 (10)	7 (7)	0.386
PICC	65 (17)	44 (16)	21 (21)	0.213
Other	1 (0)	0 (0)	1 (1)	0.092
Arterial line	24 (6)	17 (6)	7 (7)	0.727
Femoral	1 (0)	1 (0)	0	0.551
Radial	18 (5)	12 (4)	6 (6)	0.476
Axillary	0	0	0	NA
Other	4 (1)	3 (1)	1 (1)	0.959
Hemodialysis line	20 (5)	15 (5)	5 (5)	0.906
Femoral	5 (1)	4 (1)	1 (1)	0.753
Neck	15 (4)	11 (4)	4 (4)	0.962
ECMO	2 (1)	1 (0)	1 (1)	0.441
Groin	1 (0)	1 (0)	0	0.551
Neck	1 (0)	1 (0)	0	0.551
Chest	0	0	0	NA
Foley	82 (22)	55 (20)	27 (28)	0.113
Chest tube	28 (7)	19 (7)	9 (9)	0.452
Surgical drains	17 (5)	10 (4)	7 (7)	0.149
ICP monitor	4 (1)	1 (0)	3 (3)	0.025
Intra-aortic balloon pump	1 (0)	0	1 (1)	0.092
Ventricular assist device	0	0	0	NA
Any restraints	60 (16)	36 (13)	24 (24)	0.008
Wrist and/or leg	49 (13)	26 (9)	23 (23)	< 0.001
Elbow immobilizer	0	0	0	NA
Other (no description)	10 (3)	9 (3)	1 (1)	0.239
Any pressure ulcers	17 (5)	12 (4)	5 (5)	0.753
Sacral	4 (1)	3 (1)	1 (1)	0.959
Occipital	6 (2)	4 (1)	2 (2)	0.686
Heel	1 (0)	1 (0)	0	0.551
Other^*^	7 (2)	5 (2)	2 (2)	0.882

PT - physical therapy; OT - occupational therapy; HFNC - high-flow nasal cannula; CPAP - continuous positive airway pressure; BiPAP - bilevel positive airway pressure; ETT - endotracheal tube; HFOV - high-frequency oscillatory ventilation; FiO_2_ - fraction of inspired oxygen; PEEP - positive end-expiratory pressure; SBS - State Behavioral Scale; RASS - Richmond Agitation Sedation Scale; BIS - Body Image Scale; GCS - Glasgow Coma Scale; PICC - peripherally inserted central catheter; ECMO - extracorporeal membrane oxygenation; ICP - intracranial pressure.

* Includes ear, face, malleolus. The results are expressed as n (%) or the median (interquartile range).

### Therapy characteristics

By Day 3 of pediatric ICU admission, 41% of patients had a therapy session, and 90% of patients had at least one therapy session on the study day. Thirty-nine percent of patients had an order placed for a PT or OT by Day 3 of their ICU stay. Children with baseline PCPC scores of 1 (good) and 4 (severe disability) were more likely to have an order for a PT or OT placed by Day 3 in the pediatric ICU than those with mild or moderate disability. Table 4 shows the therapy characteristics by health care provider and family.

**Table 4:** Therapy characteristics

	All patient-days n = 375	PT/OT provided mobility n = 277	No PT/OT provided mobility n = 98	p value
Therapy characteristics				
PT or OT order by 9 AM on the study day	214 (57)	128 (60)	86 (40)	0.174
Days to PT or OT order	0 (0 - 5)	0 (0 - 5)	3.5 (1 - 6)	0.03
PT or OT order by Day 3 of the ICU stay	147 (39)	140 (51)	7 (7)	< 0.001
Therapy session by Day 3 of the ICU stay	153 (41)	148 (53)	5 (5)	< 0.001
At least one therapy session on the study day	337 (90)	126 (85)	211 (93)	0.014
Physical therapy	276 (74)	168 (61)	108 (39)	0.008
Occupational therapy	14 (4)	10 (71)	4 (29)	0.260
Nursing	216 (58)	146 (68)	70 (32)	< 0.001
SLP	54 (14)	24 (44)	30 (56)	0.048
Family	159 (52)	120 (75)	39 (25)	< 0.001

PT - physical therapy; OT - occupational therapy; ICU - intensive care unit; SLP - speech language pathologist. The results are expressed as n (%) or the median (interquartile range).

### Therapist-provided mobility

**Figure 1:**
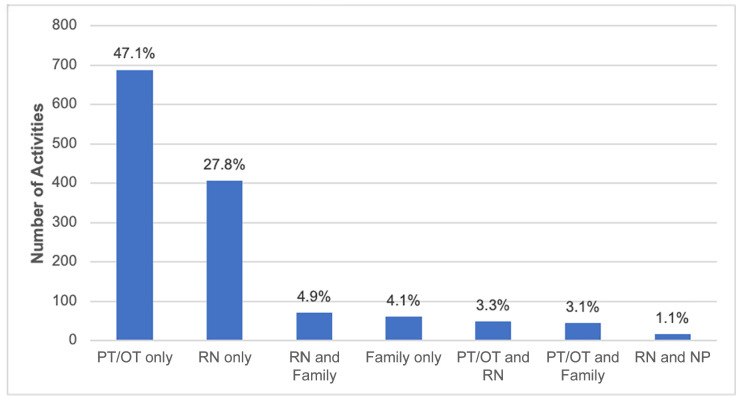
Number of activities by clinician type.

Figure 1 shows the number of activities by clinician type. The PT- or OT-provided therapy was prevalent for 74% of patients over the two study days, with 74% of all therapy sessions having a PT present, while only 4% had an OT present. Tables 3 and 4 detail the prevalence of physical or occupational therapy for demographic and clinical factors as well as for various therapy characteristics and barriers to mobility. Multivariable regression analysis showed that therapist-provided mobility was positively associated with ages 3 years and up (aOR 2.19; 95%CI 1.10 - 4.34), severe baseline disability (PCPC score of 4 *versus* 1; aOR 5.20; 95%CI 1.80 - 15.08), a nurse-to-patient ratio of 1:2 or 1:3 as opposed to 1:1 (aOR 4.97; 95%CI 2.27 - 10.89; aOR 3.84; 95%CI 1.44 - 10.25, respectively), benzodiazepine infusion (aOR 2.36; 95%CI 0.85 - 6.58), and vasoactive infusion (aOR 2.98; 95%CI 1.07 - 8.28). Factors that were negatively associated with PT- or OT-provided mobility included baseline function of a coma or vegetative state (PCPC score of 5 *versus* 1; aOR 0.52; 95%CI 0.11 - 2.47), mechanical ventilation via an endotracheal tube (aOR 0.56; 95%CI 0.19 - 1.65), urinary catheters (aOR 0.66; 95%CI 0.26 - 1.68), and central venous catheters (aOR 0.62; 95%CI 0.33 - 1.17).

PT - physical therapy; OT - occupational therapy; RN - registered nurse; NP - nurse practitioner.

### Out-of-bed mobilization

**Figure 2:**
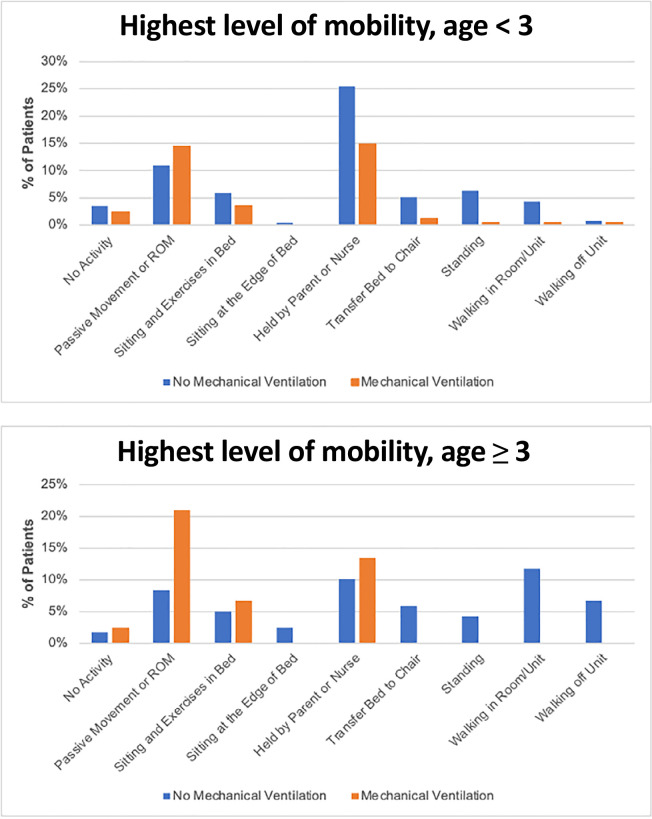
Highest level of mobility.

Figure 2 shows the highest level of mobility on the study days: 57% of patients (n = 213) were mobilized out of bed over both study days, and most (62%) of those patients were held by a parent or nurse. Of the patients who were invasively mechanically ventilated, 41% (61/148) achieved out-of-bed mobility. Out-of-bed mobility was positively associated with family presence at the bedside (aOR 3.31; 95%CI 1.70 - 6.43), mild baseline disability (aOR 2.70; 95%CI 1.23 - 5.95), and PT- or OT-provided therapy (aOR 2.86; 95%CI 1.59 - 5.12). For children 3 years and above, the presence of family by the bedside had the strongest positive association, whereas for children less than 3 years old, a PCPC score of 1 (mild disability) had the largest positive impact (Figure 2).

ROM - range of motion.

### Barriers to mobilization and safety events

**Figure 3:**
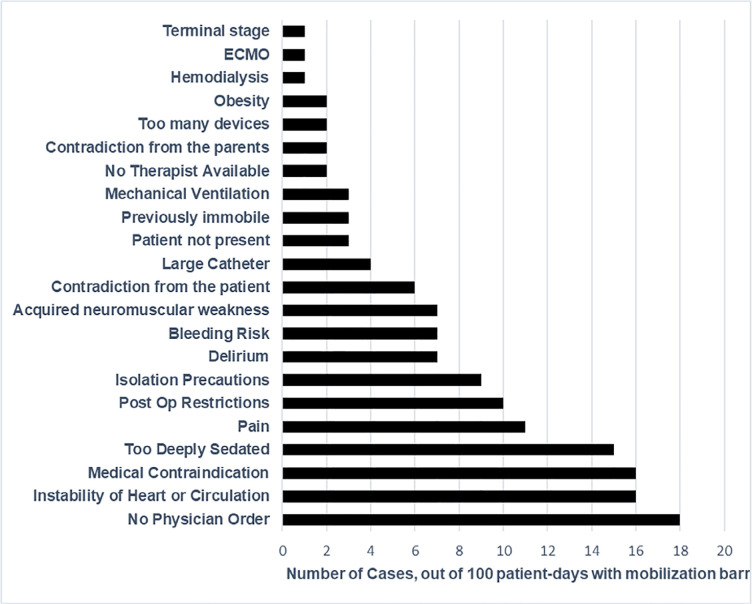
Mobilization barriers (375 patient days).

Figure 3 shows the mobilization barriers during the study days. A total of 27% of patient-days had at least one barrier to mobilization reported (n = 100). Of those, the most common barrier reported was lack of a physician order (n = 18, 5%), followed by medical contraindications (n = 16, 4%), hemodynamic instability (n = 16, 4%), and too deep sedation (n = 15, 4%). Of 1,462 mobilization activities, 43 (3%) had a potential safety event. The most common safety events were a transient decrease in O2 saturation (37%, n = 16), change in heart rate (21%, n = 9), and change in respiratory rate (19%, n = 8). Displacement of lines was not reported for any activity.

ECMO - extracorporeal membrane oxygenation.

## DISCUSSION

Our study presents the first estimates of routine mobilization practices in Brazilian pediatric ICUs, demonstrating that mobilization was quite frequent among critically ill children in our sample. Patients received therapist-provided mobility on 74% of the study days, which is roughly double the prevalence found in the USA (35%) and European (39%) studies and similar to that in Canada (80%). Physiotherapists and nurses were the most frequently involved in mobilization, and the presence of parents was strongly associated with out-of-bed mobility, highlighting important similarities and differences to PARK-PICU studies from across the globe.^(14,15,17)^ Out-of-bed mobilization in mechanically ventilated patients was significantly higher in Brazil (41%) than in Canada (36%), the USA (30%) and Europe (30%). This could be due to the presence of a PT 24/7 in most Brazilian pediatric ICUs. However, no mechanically ventilated patients were mobilized out of bed or transferred to a chair in Brazil if they were more than 3 years of age, whereas in the USA, the rate was 10%, and in Europe, it was 13%. This could have been due to limitations in nurse staffing, since older patients often require more than one person to be safely mobilized. An important result to note and potential limitation is that 41% of patients had therapy during the first 3 days (at least once) compared to 90% on the study day, which may have been due to staff awareness of the study.

The rate of potential safety events, mostly transient vital sign changes, was low (3%) despite the higher rates of mobilization in Brazil and was comparable to the USA and European studies, which ranged from 4% - 6%. Importantly, dislodgement of a device was not reported. In the US study, such dislodgement was reported in 2 of 1299 (0.15%) mobilization events, whereas in the European study, endotracheal tube dislocation occurred only once.^(14,15)^

The main barriers to mobility in Brazil were “no physician order” (p < 0.001), cardiovascular instability (p < 0.001), and “no therapist available” (p = 0.017), which was consistent with other studies. We observed that, despite the 39% rate of physician orders, a much higher rate of mobilization was performed irrespective of a lack of physician orders. We attribute this finding to Brazilian PTs’ practice of independently evaluating patients and providing mobilization.

Interestingly, in the USA, intubation was the major barrier, followed by urinary catheterization, whereas in Brazil, similar to Europe, the main barriers were cardiovascular instability, oversedation and medical contraindication.^(14,15)^ An important difference between these international regions is that the lower nurse to patient ratio of 1:2 or 1:3 as opposed to 1:1 could be associated with a lower rate of mobilization by the nurse alone in Brazil (27.8%) compared to North America (48%) and Europe (46%).^(14,15)^

The Brazilian Guidelines for Early Mobilization in Intensive Care Unit were published in 2020, specifically focused on adults.^(9)^ Pediatric guidelines are still lacking; however, 45% of pediatric ICUs have their own early mobilization protocol, in stark contrast to other countries across the globe.^(18)^ According to a systematic review, the implementation of multidisciplinary protocols seems to be a feasible tool for the promotion of early mobilization in pediatric intensive care.^(19)^ Thus, it is a sign that it is time to join efforts to publish Brazilian pediatric guidelines. There is a paucity of PICU mobilization therapy data from lowand middle-income countries with which to compare our data. Hence, this study is a cornerstone in establishing standards of care in Brazilian pediatric ICU practice and provides a model for how early mobility can be optimized and sustained even with limited resources. There is a regulation (RDC [*Resolução da Diretoria Colegiada*] 7) dating from 2010 that requires a PT for 18 hours a day over 3 shifts in pediatric ICUs.^(20)^ However, many pediatric ICUs in Brazil have already worked with 24/7 physiotherapists, and 4 states already have a regulation with that recommendation. A national regulation is under consideration to require the presence of PTs 24/7 in all ICUs.^(21)^ It is important to note, however, that physiotherapists in Brazil often fulfill the duties of both respiratory therapy and occupational therapy, which is in contrast to models in the United States with designated staff for each of those roles, for example. We did not address the workload of physiotherapy staff in this study; however, our findings demonstrate that mobilization is not negatively impacted despite the multiple responsibilities of physiotherapists. However, the needs of the youngest children, who are possibly the most vulnerable population, need to be urgently addressed. We found that, similar to patients in Europe and North America,^(14,15)^ these patients are less likely to receive mobility therapy, which is usually facilitated by occupational therapists for habilitation during active physical and neurocognitive development.

Our study has several important limitations. First, the centers that accepted the invitation to participate in the study may have had more interest in research and early mobilization, potentially biasing the results to overestimate mobilization practices. Second, mobility assessments were unblinded, which may have led to greater mobility delivery on the study days because the staff was aware of the study, possibly leading to the Hawthorne effect.^(22)^ Third, we could not report whether a patient met the criteria or had contraindications to medically mobilize or get out of bed. Finally, it is possible that the results of this study are not generalizable to all Brazilian pediatric ICUs. However, there has never been a study of this magnitude or with this number of centers that has focused on early mobility in pediatric ICUs.

## CONCLUSION

In this point prevalence study, children from this sample in Brazil received mobilization on 74% of the study days, which is roughly double that found in USA and European studies. Physiotherapists are the most frequent providers of mobilization, confirming that their frequent and consistent presence in pediatric intensive care units is instrumental to establishing a culture of mobility for critically ill children. Family presence was high, which was positively associated with out-of-bed mobilization. Further longitudinal studies should confirm whether Brazilian pediatric intensive care unit mobilization practices may be a model for other countries to consider in guiding health care policies, implementing protocols, and designing new studies.

Nelson Kazunobu Horigoshi - *Hospital Infantil Sabará*, São Paulo, SP; Graziela de Araújo Costa - *Hospital Sírio-Libanês*, São Paulo, SP; Taísa Roberta Ramos de Castilho - *Hospital Anália Franco Rede D’Or São Luiz* and *Hospital Beneficência Portuguesa*, São Paulo, SP; Paula Peres Domingues Peron - *Instituto de Criança, Hospital das Clínicas, Universidade de São Paulo*, São Paulo, SP; Walter Perez Scaranto - *Hospital Municipal Carmino Caricchio*, Tatuapé, São Paulo, SP); Daniela Nasu Monteiro Medeiros - *Hospital Municipal Dr. Moyses Deutch*, M’Boi Mirim, São Paulo, SP; Toshio Matsumoto - *Hospital Municipal Infantil Menino Jesus*, São Paulo, SP; Carlos Gustavo de Almeida - *Hospital Assunção Rede D’Or São Luiz*, São Bernardo do Campo, SP; Felipe Rezende Caino de Oliveira - *Grupo de Apoio ao Adolescente e à Criança com Câncer, Instituto de Oncologia Pediátrica*, São Paulo, SP; Marcelo Barciela Brandão - *Universidade de Campinas*, Campinas, SP; Fernanda Lima-Setta - *Instituto Fernandes Figueira*, FIOCRUZ, Rio de Janeiro, RJ; Arnaldo Prata-Barbosa - *Hospital Copa D’Or, Hospital Quinta D’Or, Hospital Caxias D’Or, Hospital Rios D’Or, Hospital Oeste D’Or* and *Hospital Real D’Or*, Rio de Janeiro, RJ); Glaciele Nascimento Xavier - *Instituto de Cardiologia do Distrito Federal*, Brasília, DF; Livia Barbosa de Andrade - *Hospital Esperança*, Recife, PE; Agda Ultra de Aguiar - *Hospital de Base do Distrito Federal*, Brasília, DF; Marcos Paulo Galdino Coutinho - Hospital Otávio de Freitas, Recife, PE; Roberta Esteves Viera de Castro - *Hospital Universitário Pedro Ernesto, Universidade do Estado do Rio de Janeiro*, Rio de Janeiro, RJ); Glazia André Landy; *Instituto de Tratamento do Câncer Infantil* - São Paulo, SP; Suzana Lopes Bonfim Balaniuc - *Hospital Universitário Maria Aparecida Pedrossian*, Campo Grande, MS; Ricardo Silveira Yamaguchi - *Hospital da Luz*, São Paulo, SP.
